# Introducing an interactional approach to exploring facilitation as an implementation intervention: examining the utility of Conversation Analysis

**DOI:** 10.1186/s43058-020-00071-z

**Published:** 2020-11-04

**Authors:** Sarah C. Hunter, Jessica A. Young, Michael T. Lawless, Alison L. Kitson, Rebecca Feo

**Affiliations:** 1 College of Nursing and Health Sciences, Flinders University, Sturt Road, Bedford Park, South Australia 5042 Australia; 2 Caring Futures Institute, Flinders University, Sturt Road, Bedford Park, South Australia 5042 Australia; 3 Health and Rehabilitation Sciences Program, Faculty of Health Sciences, Western University, London, Ontario N6A 3 K7 Canada

**Keywords:** Evidence-based practice, Facilitation, Knowledge Translation, Conversation Analysis, Interaction, i-PARIHS, Implementation

## Abstract

**Background:**

The widely adopted integrated-Promoting Action on Research Implementation in Health Services (i-PARIHS) framework identifies *facilitation* as a ‘core ingredient’ for successful implementation. Indeed, most implementation scientists agree that a certain degree of facilitation is required to translate research into clinical practice; that is, there must be some intentional effort to assist the implementation of evidence-based approaches and practices into healthcare. Yet understandings of what constitutes facilitation and how to facilitate effectively remain largely theoretical and, therefore, provide scant practical guidance to ensure facilitator success. Implementation Science theories and frameworks often describe facilitation as an activity accomplished in, and through, formal and informal communication amongst facilitators and those involved in the implementation process (i.e. ‘recipients’). However, the specific communication practices that constitute and enable effective facilitation are currently inadequately understood.

**Aim:**

In this debate article, we argue that without effective facilitation—a practice requiring significant interactional and interpersonal skills—many implementation projects encounter difficulties. Therefore, we explore whether and how the application of Conversation Analysis, a rigorous research methodology for researching patterns of interaction, could expand existing understandings of facilitation within the Implementation Science field. First, we illustrate how Conversation Analysis methods can be applied to identifying what facilitation looks like in interaction. Second, we draw from existing conversation analytic research into facilitation outside of Implementation Science to expand current understandings of how facilitation might be achieved within implementation.

**Conclusion:**

In this paper, we argue that conversation analytic methods show potential to understand and refine facilitation as a critical, and inherently interactional, component of implementation efforts. Conversation analytic investigations of facilitation as it occurs in real-time between participants could inform mechanisms to (1) improve understandings of how to achieve successful implementation through facilitation, (2) overcome difficulties and challenges in implementation related to interpersonal communication and interaction, (3) inform future facilitator training and (4) inform refinement of existing facilitation theories and frameworks (e.g. i-PARIHS) currently used in implementation interventions.

Contributions to the literature

- Interactional and collaborative behaviours are not only important in facilitation, but they constitute it.
- Successful facilitation hinges upon how effectively facilitators communicate with stakeholders. In this way, a ‘successful’ facilitator can be distinguished by their ability to deploy communicative resources in deliberate, responsive and creative ways to achieve key processes and outcomes, such as providing feedback, challenging shared assumptions, or developing new perspectives.
- We propose that conversation analytic methods can be applied to the analysis of facilitation in practice, to establish an evidence base of communication practices and patterns pertinent to facilitation, which can be used for practical, educational and theoretical purposes.

## Background

The field of Implementation Science seeks to provide various approaches, frameworks and theories to inform systematic and successful implementation of research into practice [1]. These approaches, frameworks and theories can assist in teasing out why some implementation efforts are more successful than others, and thereby increase the likelihood of future implementation success [2]. One of the most widely used of these frameworks is the integrated-Promoting Action on Research Implementation in Health Services (i-PARIHS [3]). i-PARIHS is a conceptual framework designed to represent the dynamic interplay of factors that influence the success of implementation [3]. As a point of difference from other implementation frameworks, i-PARIHS identifies *facilitation* as the core ingredient for successful implementation. Specifically, successful implementation is seen to result from effective facilitation of an innovation with the intended ‘recipients’ (i.e. the staff, services and patients that will be directly involved in and affected by the implementation process) in their contextual setting [3]. The proposition of i-PARIHS can be seen in Table 1.

**Table 1:** Overview of i-PARIHS

i-PARIHS	
SI = Fac^*n*^ (*I* + *R* + *C*)	
SI = Successful implementation Fac^*n*^ = Facilitation *I* = Innovation *R* = Recipients (individual and collective) *C* = Context (inner and outer)	

Within the framework, facilitation is conceptualised as both a dedicated role (‘being’ a facilitator) and set of actions (‘doing’ facilitation) [3]. Given the centrality of facilitation to the framework, an accompanying i-PARIHS facilitation guide was developed, which includes a Facilitation Checklist and a Facilitator’s Toolkit [4]. The guide raises various questions and issues that facilitators need to consider in order to guide successful implementation. Examples of these questions include ([4], p. 54):

- Do recipients of the intervention perceive the proposed change as valuable and worthwhile?
- Is the change consistent with their existing values and beliefs?
- Are there individuals within the organisation where the intervention is being implemented who function as local opinion leaders? Will they be supportive or obstructive in terms of introducing the proposed change?

The i-PARIHS framework outlines how addressing these questions can support successful implementation and inform facilitation activities. What remains unknown, however, is how facilitators’ consideration of/responses to these questions translate to practice. In this debate article, we argue that Implementation Science would benefit from adoption of research approaches that acknowledge the interactional nature of facilitation and which can provide evidence on how facilitation can be accomplished in situ.

## Objective

We propose the application of conversation analytic methods to study, systematically and rigorously, i-PARIHS facilitation in situ (i.e. as it occurs naturally in conversations between facilitators and recipients) and demonstrate the potential of this application for providing the operational detail that the i-PARIHS framework currently lacks. First, through a small number of illustrative examples, we demonstrate how Conversation Analysis methods can be applied to identify what facilitation ‘looks like’ in interaction. Second, we draw from existing conversation analytic research into facilitation *outside* of Implementation Science to expand current understandings of how facilitation might be achieved *within* Implementation Science. Finally, in the discussion, we consider the utility and potential practical outcomes of applying Conversation Analysis methods to i-PARIHS facilitation data (i.e. recorded interactions amongst facilitators and recipients).

### The facilitation conundrum

i-PARIHS, and other implementation theories that move beyond the notion of a ‘linear pipeline’ of implementation, hold an underlying philosophy that implementing research into healthcare practice is complex and unpredictable [4]. Such complexity, in practice, can translate to difficulties ‘fitting’ the intervention as envisaged to the context of its implementation, resulting in poor implementation outcomes. These difficulties indicate that more focused and active efforts are needed to ‘facilitate’ the practical changes required in implementation initiatives. Hence, many theories and frameworks in the field of Implementation Science recognise the need for interactive, context-responsive implementation processes [5]. The field of Implementation Science as a whole, therefore, appreciates the importance of facilitation to tailoring and actively promoting the uptake of research into clinical practice.

The increasing popularity, and documented effectiveness of, facilitation can be attributed to its flexibility in terms of enabling implementation scientists (and anyone implementing evidence) to adopt creative problem-solving strategies to address barriers as they emerge in the local implementation context. However, harnessing this flexibility when employing a facilitation approach requires an understanding of the complex roles and activities of a facilitator. Given this, Harvey and Kitson [4] outline how the facilitator role evolves as he or she develops and refines their skillset, with facilitators beginning as a ‘novice’ and working their way through to an ‘experienced’ and finally an ‘expert’ facilitator. Novice facilitators are often paired with an experienced and/or expert facilitator to provide support and mentoring [4]. In addition to the differences between novice, experienced and expert facilitators, there are also differences between internal and external facilitators [3]. External facilitators with no pre-existing connections with stakeholders in the implementation setting will need to rapidly form relationships to tap into tacit knowledge about the local context and recipients. By comparison, an existing staff member might take on the facilitator role in their own workplace, making them an internal novice facilitator, who might be familiar with the local context but have to reframe their role from one of ‘doing’ to one of ‘enabling’.

The complexity of enacting facilitation within implementation projects has resulted in criticisms that the i-PARIHS framework lacks operational detail on *how* to facilitate in practice, and specifically, in terms of the interpersonal and communicative dimensions that are at the core of facilitation. What remains unknown and, to date, not adequately explored is *how* successful or skilled facilitators apply the right facilitation strategies, at the right time, with the right people, in the right way. Even though the i-PARIHS framework outlines the central attributes and personality characteristics that make certain individuals better suited for facilitation (i.e. patient, resilient, pragmatic, curious) ([4], pp. 73–76), what is required to move this understanding forward is a detailed and rigorous exploration into how these ‘experienced’ or ‘expert’ facilitators accomplish what has commonly been considered an artful skill. In addition, the i-PARIHS framework developers recognise that there is a need to further develop and refine the evidence base around facilitation and to provide practical guidance for facilitators [3].

Facilitation, whilst a central component of the i-PARIHS framework, is not unique to the field of Implementation Science and implementation frameworks and theories. Facilitation is also commonly used as a quality improvement strategy in primary care practices and this is known as practice facilitation [6]. This facilitation strategy is described as a multifaceted approach to implementing evidence into practice involving trained individuals who enable others to improve their practice through a multitude of activities (e.g. stakeholder engagement, provider feedback, education [7];). A recent study of an implementation initiative in the USA found that practice facilitation can have positive impacts at a large-scale (i.e., at the national level) [8]. However, success at this scale requires the appropriate infrastructure and support [8].

Facilitation is widely used and even though practice acilitation and i-PARIHS have emerged as distinct approaches (i.e. practice facilitation as a quality improvement strategy used in primary care practices and i-PARIHS as an implementation framework often used for complex interventions), there is significant overlap in how they appreciate facilitation is the key mechanism to enact change. A recent study highlighted the similarities between practice facilitation and i-PARIHS and the ways in which their respective conceptualisations of facilitation are complimentary [9]. Therefore, whilst there may be a vast literature examining the use of facilitation in a variety of environments, an exploration into i-PARIHS informed facilitation as an inherently interactive activity that requires creativity, sensitivity and reflexivity has application beyond those using the i-PARIHS framework.

### Understanding facilitation from an Implementation Science lens

There is an increasing, although limited, amount of published implementation studies that seeks to explicate their facilitation approach. These studies provide detail about how facilitators put the theoretical concept of i-PARIHS facilitation into practice within their unique context. Most of these studies have employed qualitative self-report or observational methodologies to understand, in greater detail, the experiences of facilitating implementation. This section will discuss a few examples of how facilitation has been described in the Implementation Science literature. What this will demonstrate is that, despite differences in innovation, recipients and context, these studies shared a conceptualisation of facilitation as an interactive and ‘hands-on’ achievement that requires continual communication and conversation between facilitators and recipients.

A study in Norway utilised ethnography to explore the facilitation of a workplace education intervention in a nursing home, using 2-day facilitator-led seminars for staff followed by five 1-h facilitation sessions over 6 months to tailor the educational intervention to the nursing home context [10]. In an exploration of the facilitation processes, the staff reported that they appreciated the facilitator being able to encourage them to improve their care as well as to enable them to work together as a team.

Another example of research exploring facilitation experiences includes a study in the UK that utilised interviews and field notes to examine the implementation of person-centred assessment and support in palliative care, using a model of internal and external facilitators, where all internal facilitators supported their local sites and the external facilitators provided monthly ‘peer support’ sessions to troubleshoot any barriers [11]. This study described how facilitation was crucial to motivating and supporting the use of the intervention within the specific context of implementation. This study also highlighted that, in order for facilitation to successfully support implementation of the intervention, facilitators had to communicate effectively with, and enable collaboration amongst, the intended recipients.

A case study in Australia interviewed clinicians who took on the role of local, novice facilitators in a nutrition implementation project to advance understandings of the facilitation process [12]. This study highlighted that internal facilitators were crucial for building trust and relationships amongst clinicians and bringing together multidisciplinary teams. Bringing people together was shown to occur primarily in the context of formal meetings, but also through informal discussions or ‘check-ins’, both of which typically involved one or more facilitators and at least one recipient. The facilitator’s role of bringing recipients together through formal and informal channels was described as a continuous activity, which might also be framed as ‘keeping the team together’.

A final example of previous research exploring facilitation experiences includes a doctoral thesis that explored facilitation within a neonatal intensive care unit [11]. In this research, facilitation was identified as crucial for bringing people together. This was typically achieved through the facilitator ‘reaching out’ to recipients. Building connections and trust between facilitator and recipients was deemed to be of great importance and was framed as largely the responsibility of the facilitator. This work also characterised the overall success of the implementation process as hinging on ‘the relationship, or lack of a relationship, that the facilitator developed with their assigned collaborative team.’ (pp. 88–89). Further, in the same study, recipient collaboration (e.g. shared decision-making and creating team action plans) was argued to be achieved, in part, by facilitators encouraging recipients to reflect on (what?) to help find potential solutions. Examples include questions related to the innovation in context, such as “How do you make a change in your organization?” and “What are your challenges in doing it the way that the recommendations are?” (pp. 80–81).

In sum, this section has discussed how facilitation is currently described in the Implementation Science literature, and we have begun to demonstrate how facilitation occurs through communication and interaction. Further, the effectiveness and quality of facilitation practice appears to be largely contingent upon how facilitators manage these conversations. In this way, a ‘successful’ facilitator can be distinguished by their ability to deploy communicative resources in deliberate, responsive and creative ways to achieve key processes and outcomes, such as providing feedback, challenging shared assumptions or developing new perspectives [3].

As this section demonstrates, existing research on facilitation has typically utilised observational methodologies and interviews to investigate *what* facilitators and recipients do (Table 2). Whilst this body of work provides practical and useful insight into what facilitation involves, additional research, utilising a different methodological approach, is required to offer guidance on *how* facilitators and recipients might achieve certain actions (e.g. how does one *provide feedback* successfully? How does one *challenge assumptions* in a way that benefits the implementation efforts? How does one go about *developing new perspectives* in team meetings?). Therefore, there is a need to further explore how facilitation can successfully be achieved in situ (i.e. ‘on the ground’; in interaction amongst facilitators and recipients). Below we introduce one methodology that can be used to address these questions.

**Table 2:** The ‘what’

Facilitator goals are: ­ reaching out ­ bringing a team together ­ keeping the team together ­ presenting information ­ guiding the process ­ fostering connection and trust ­ enabling collaboration ­ encouraging reflection ­ making decisions together ­ creating team action plans ­ finding solutions ­ prompting reflection	

### Introducing Conversation Analysis

A variety of methodologies have emerged over the past few decades in order to examine social phenomena such as interaction. Ethnomethodology is an example of such an approach and was the first to outline the importance of interaction for examining how people make sense of the social world [14–16]. Many methodologies have since emerged that highlight the significance of language and interaction. For example, discourse analysis is an approach that focuses on the various ways in which language is used to construct and accomplish social actions [17–19]. Another interactional approach is the Roter Interaction Analysis System (RIAS), a method for coding medical interactions [20]. However, in this paper, we have chosen to focus specifically on one interactional approach, Conversation Analysis [21]. This methodology has been selected as it places emphasis on induction and data-internal validity, as well as its focus on the sequential nature of conversation and its ability to focus on detailed and fine grain analysis (i.e. the focus on tone, pitch, overlapping talk), each of which is described in more detail below.

Conversation Analysis is a methodological approach that emerged from the writings of sociologist, Harvey Sacks, and his colleagues, Gail Jefferson and Emanuel Schegloff, who pioneered methods for studying people’s use of language in everyday life. Conversation Analysis is concerned with examining the ways in which social actions (e.g. agreement, disagreement, complaining, requesting, inviting) are routinely achieved through the deployment of specific verbal and non-verbal resources in conversation [22]. A basic premise of Conversation Analysis is that conversation is action-oriented and that actions are achieved in a sequential and orderly sequence of conversational turns. The focus of analysis is on how participants, in situ, orient to the actions that are achieved through conversation and how these orientations are made visible in speakers’ turns, as a conversation unfolds.

In order to undertake Conversation Analysis, audio or audio-visual recordings of naturally occurring conversations between people are required. These recordings are generally designed to be minimally intrusive so as to capture ‘what would go on whether or not the research were in progress’ ([23], p. 3). Recordings are then subjected to repeated listening and/or viewing and fine-grained transcription. This transcription typically involves using conventional Conversation Analysis notation to represent features of speech delivery and intonation (e.g. volume, pitch, vocal quality) as well as temporal and sequential relationships between speakers and their utterances (e.g. overlapping talk, silences [24];, see Additional file 1). Close examination of collected conversations, and detailed transcripts thereof, is undertaken to generate descriptions of typical features of communication sequences. Once patterns and practices have been named and described in detail—typically through reference to numerous illustrative data extracts—empirical findings are then interpreted to generate understandings about the structure, functioning and outcomes of different communication practices [23].

Conversation Analysis, much like other interactional approaches, has the advantage of being able to examine what is occurring at the time of interaction, as opposed to relying on retrospective participant self-report and reflection (as is needed in interviews). However, unlike many other interactional approaches, such as Discourse Analysis, Conversation Analysis provides the added advantage of allowing us to focus not only on the facilitator and what they say, but on how different actions are achieved interactionally and sequentially between facilitators and recipients. That is, how conversational sequences (multiple turns-at-talk by different speakers) unfold and the interactional consequences of different turns-at-talk (e.g. whether advice is accepted or rejected and the outcomes of this acceptance/rejection for the unfolding interaction and accomplishment of facilitation goals). Whilst the Roter Interaction Analysis System (RIAS) also provides the opportunity to analyse sequential conversation, it has, to date, focused on medical interactions and was not designed to be used to analyse interactions involving multiple speakers (e.g. one facilitator and multiple recipients).

Conversation Analysis research has generated a substantial body of knowledge about the structure and functioning of communication practices in a variety of contexts, including healthcare delivery [25–27], mediation hearings [28, 29], legal interactions [30, 31], and informal family conversations [32, 33]. Although numerous conversation analytic studies have collected and analysed interactional data in organisational settings and reported on communication practices germane to facilitation, the approach has predominantly been developed and applied by academics working in the domains of linguistics, social psychology, and sociology. As a result, Conversation Analysis as a methodological approach has, to date, been underutilised by those working in Implementation Science or health services research. As such, health care practitioners, policymakers, and educators have had limited access to the practical knowledge that can be generated from conversation analytic research. However, before undertaking any Conversation Analysis research that examines recordings of implementation facilitation meetings, we argue it is important first to understand the potential utility of Conversation Analysis in this space.

### Applying Conversation Analysis methods to understanding facilitation in practice: an illustrative example

To demonstrate the utility of Conversation Analysis in informing and illuminating facilitation practices, we will explore one study in detail, as a case example, and provide illustrative extracts^1^. The study, by Franco and Neilsen [34], used Conversation Analysis to examine how facilitator communication shaped work group interactions (across university, business, and local government contexts). The authors found that by using three distinct types of conversational ‘formulations’ (i.e. where one speaker, the facilitator, summarises the talk up until that point), facilitators enabled collective sense-making amongst participants. The formulations described were (1) formulations that encourage reflection, (2) formulations that facilitate action, and (3) collaboratively produced formulations. For example, when encouraging reflection, facilitators commonly used a specific type of formulation; a *so-*prefaced question, such as ‘so when you say X, how does that relate to Y’. This formulation functioned to identify the ‘gist’ of a prior conversational turn and encourage participants to reflect further on the idea or topic put forth within that turn. Extract 1 demonstrates how a facilitator (Sandro) deploys the formulation over several turns (lines 1–25, in bold).

**Extract 1:** Franco and Nielsen ([34], p. 744)

01	SAN:	↓ye:s **so**
02		((sits up, leans back, looks at Joe, and starts gesturing))
03		((Jean looks at Sandro))
04		is that (0.3)
05	JOE:	((looks at screen))
06	SAN:	Joe **when you say this**
07		((points at the screen))
08	SAN:	balan↓cing portfolio
09		((looks back at Joe)) (.) of ↓ac↑tivities
10		((Callum, Liz, Matt, Gerald, Jean look at screen))
11	JOE:	yeah
12	SAN:	is an issue (0.5)
13		((looking at screen, pointing))
14		which (0.5)
15	SAN:	will achieve
16		((looks at Joe and takes down his hand))
17		higher margins
18		(0.7)
19	JOE:	yes
20	SAN:	↑**how does that**
21		((looks and points at the screen))
22		↓**relate to**
23		((looks back at Joe))
24		((Alfie looks at the screen))
25	SAN:	**the previous materi**↓**al**

Here, the facilitator’s (Sandro) formulation is designed to encourage the participant to elaborate on a previous part of the interaction (a discussion on balancing portfolio of activities), as evidenced by the beginning of his formulation on line 6: ‘when you say this’. The facilitator repeatedly uses his gaze (lines 2, 9, 16, 23, 25) to indicate the intended recipient of his formulation (Joe) and to prompt him to respond. The facilitator also gestures to a display screen as a mutual point of reference (lines 5, 7, 13, 21), helping to orient Joe to the part of the conversation on which the facilitator is attempting to encourage further reflection.

A second category of formulations commonly observed during the closing phase of the workshop, known as action-oriented formulations, was used to reach a conclusion or propose a future course of action. In Extract 2, we observe how a different facilitator (Alfie) seeks confirmation from workshop participant Jean for a course of action (line 4, in bold) previously proposed by workshop participant Joe:

**Extract 2:** Franco and Nielsen ([34], p. 747)

01	JEA:	°↑Yeah:° (0.5)
02		((nodding and looking across table))
03	JOE:	(?°we’re getting around an understanding°?) (0.5)
04	ALF:	**So you’re saying you’ll [do it?]**
05		((Looking intently at Jean))
06	JEA:	(((looking at Alfie))
07	JEA:	[yeah] >well that’s what< I:
08		((points at herself with both hands))
09	JEA:	put down
10		((pointing at screen))
11	ALF:	↑That’s what you’re gotta do:=
12		((looking intently at Jean))
12	MAT:	= (? °it’s admirable [to see?]°)
13	JEA:	[ yeah ]
14		((touches chest with both hand fingers, and then moves hands away from chest with open hand palms))
15	ALF:	((pointing to Jean))
16		We ↑have an ↑ac[tion ],
17	?:	[(?she said?)]
18	?:	((cough))
19	SAN:	Have an action [ yeah ]?
20		((looks at laptop screen))
21	?:	[>°we got°<]
22	?:	(?int(h)ensive /int(h)ension?)
23	JEA:	↑yeah, ((smiles))

Here, the facilitator Alfie uses a *so-*prefaced question (“So you’re saying you’ll do it?”, line 4) whilst specifically looking to Jean for uptake (i.e. a response). In doing so, he formulates the future action as a logical upshot (i.e. consequence) of the immediately preceding conversation. In line 11, the facilitator recycles his attempt to solicit confirmation of the action, again addressing Jean directly with his gaze, prompting her to confirm. Following confirmation from Jean (line 13), the facilitator then points to Jean (line 15) and seeks further confirmation that an action has been agreed (line 16). Following this, the second facilitator (Sandro) recycles the first facilitator’s earlier action-formulation from line 16 in an almost identical repetition (“Have an action [yeah]?” line 19). By means of formulation and gesture, then, the facilitators work together to attribute accountability for the action to Jean, who confirms with a smile (line 23).

In summary, these two examples demonstrate the significant amount of interactional work required on the part of facilitators to enable collaborative action. The examples demonstrate that we cannot focus simply on the facilitator alone to understand facilitation. Instead, we need to focus on how goals are achieved interactionally and sequentially among the facilitator and the recipients. Through this, we demonstrate how Conversation Analysis can enable us to understand what effective facilitation ‘looks like’ in practice: that is, how people successfully facilitate an intervention in and through conversation. More broadly, these examples have showcased the potential value of Conversation Analysis in demonstrating how participants accomplish goals in real time, as well as demonstrating the finer nuances of interaction within the context of facilitation.

### Conversation analytic contributions to understanding facilitation

Although ‘formulations’ are the focus of Franco and Neilson’s [34] analysis, these are just one of many interactional ‘tools’ or resources facilitators and recipients might use in the facilitation process. We turn now to a summary of additional examples from the Conversation Analysis literature of the interactional resources used to ‘do facilitation’ in interaction.

Conversation Analysis as a research methodology has been applied to better understand facilitation in fields outside of Implementation Science (e.g. business, management science, and organisational psychology). Here, we draw from studies conducted in organisational or business settings (as opposed to, e.g. medical interaction or classroom interaction) because of their contextual relevance in terms of participant roles and demographics (e.g. team leaders, workshop facilitators), institutional tasks, reported interactional phenomena, and implications for implementation [35–37]. Studies employing Conversation Analysis in organisational and business settings have reported on a range of social and interactional phenomena relevant to facilitation, such as proposing future actions [35], and facilitating common agreement/achieving consensus on issues of significance [36, 37]. A summary of what these studies have identified about *how* facilitation occurs is summarised in Table 3.

When focusing on proposals for future action, Asmuß and Oshima [35] examined sequential patterns following a proposal (such as, ‘shall we…’) and whether these proposals (typically presented in the form of questions) are accepted or rejected. The analysis showed that how the ‘proposer’ expressed the question (i.e. tone, pitch) influenced the outcome (acceptance/rejection) because it displayed different degrees of entitlement to be asking the question. Overall, what this study makes clear is that individuals do not simply respond to a question; they respond based on how the question is asked, including the implications underpinning the question (such as whether someone displays authority to ask such a question of someone). In Barnes’ [36] examination of facilitating common agreement in meetings, it was identified, via Conversation Analysis, that the meeting chair draws on what is deemed a ‘glossing practice’ to acknowledge previous discussions to confirm a consensus agreement, without going into detail or specifics of this previous discussion (i.e. they ‘gloss’ over it). Through this interactional achievement, the meeting chair can establish, record, and preserve agreement. Finally, Wasson [37] examined how consensus-oriented decision making is achieved in meetings when participants are focused on agreement for issues of significance. Through Conversation Analysis, this study identified that, following an initial proposal, agreement can take place, where participants align with the proposers’ statement. However, when disagreement immediately follows an initial proposal, an interesting display of information sequences and joking sequences followed to manage the disagreement. Information sequences involved participants requesting more information about the proposal and joking sequences included making light of the disagreement to repair relationships between the participants and ease tension, particularly in the case of numerous disagreement sequences. This study demonstrated how these various sequences were drawn on at different times over a meeting to accomplish a consensus decision when instances of disagreement occurred.

**Table 3:** The ‘how’

Facilitators achieve desired aims by: - displaying low levels of entitlement when proposing future action - glossing preceding talk to demonstrate collective agreement and close the ‘business-at-hand’ to move onto the next topic - working through agreement, disagreement, information and joking sequences to work toward consensus decision making	

### Limitations of Conversation Analysis

Conversation Analysis, like many methodologies, is not without its limitations. One of which being how it handles context. Facilitation is an effective and widely used implementation strategy, as it is responsive and flexible to the context in which it is occurring. However, the notion of context is defined differently within the Conversation Analysis tradition. Generally, Conversation Analysis treats context in a very narrow way, specifically, it views conversation as ‘context-free’ [38]. It is context-free in that it views the mechanics of interaction (i.e. turn-taking, tone, pitch, overlap) as situated within the immediate and local sense of the utterance’s context, without overt consideration of the participants’ cultural and historical context. The consideration of broader context, such as where a conversation is taking place and the characteristics of the speakers (i.e. age, gender, race) is not appropriate analytically, unless it presents itself in the interaction; that is, unless it becomes a topic for conversation between facilitator and recipients. Therefore, through such an analysis, important contextual influences might be overlooked. This stands in contrast to ethnography and forms of Discourse Analysis, including those influenced by post-structuralist theory, taking into account the broader cultural and historical production of social meaning (e.g. [17–19]), which have the power to regulate and discipline the positions that are available for people to occupy.

This micro-level analysis that minimises the cultural and historical context in which a conversation is occurring is what is considered a strict, purist or ‘Schegloffian’ version of Conversation Analysis. This treatment of context has led to debates with one example being by Moerman [39] who celebrates Conversation Analysis’ methodological rigour but criticizes it for its overly rigid treatment of context. For this reason, Moerman and other researchers argue for Conversation Analysis to be conducted in conjunction with other methodologies that allow for contextual features to be considered (i.e. observation, interviews). Further, there is a body of literature that undertakes Conversation Analysis whilst also attending to broader contextual issues, such as the work of Celia Kitzinger [40–42] and Susan Speer [43–45] on gender, feminism and Conversation Analysis. This demonstrates the capacity for researchers to utilise Conversation Analysis in a way that captures the importance and complexity of broader contextual factors.

Another potential limitation of Conversation Analysis is the need to video and audio record interactions for analysis. The facilitation literature, as outlined in this paper, demonstrates that formal facilitation interactions often are complemented by informal, ad hoc interactions among recipients and facilitators [12, 13]. Recording these less-formal interactions might prove challenging, given their ‘impromptu’ nature. Thus, although recordings of formal facilitation meetings will likely prove useful to understanding facilitation as an interactional achievement within groups, we are limited in our ability to generalise these findings to related, but contextually different and often dyadic (one-on-one), informal interactions.

Hence, this is why we propose Conversation Analysis as an *additional* methodology to examine facilitation within implementation, to complement current understandings of the facilitation process. Conversation Analysis might have particular benefit when utilised in a mixed-method approach. Conversation Analysis, with its emphasis on induction and data-internal validity, could be performed in conjunction with observation, questionnaires, interviews, and document analysis to enable data-external validation, serving as a form of triangulation and enabling a more macro interpretation of facilitation that incorporates and respects context.

## Discussion

Facilitation has been identified as a crucial component for the successful implementation of evidence into practice. Yet, there remains uncertainty around what successful facilitation looks like [3]. The purpose of this paper was to highlight the hitherto under-explored applicability of Conversation Analysis to the field of Implementation Science and demonstrate its potential utility in understanding and explicating facilitation as an interactional achievement.

Conversation Analysis allows for an exploration into the specific communication practices that constitute and enable facilitation. These practices remain inadequately defined in the Implementation Science literature, limiting the success of implementation efforts that necessarily require collaboration and interaction among stakeholders. Therefore, the practical implication of applying Conversation Analysis to facilitation research in the implementation space is that we can:

1. Improve understandings of how to achieve successful implementation through facilitation, such as by identifying specific communication practices that are instrumental in the enactment of key facilitative tasks;
2. Overcome interactional difficulties and challenges in implementation, such as by providing facilitators with interactional strategies to manage conflict or resistance; and
3. Begin to understand, with specific interactional evaluation techniques, the fidelity of facilitation and the appropriate dose.

In turn, this improved understanding can inform future facilitation training. Specifically, the findings of conversation analytic research focussing on facilitation could contribute to the development of practical resources designed to improve facilitators’ repertoire of skills by deepening understandings of different conversational formulations and their interactional consequences. These resources could take the form of communication training materials based on recordings of actual interactions, in contrast to the use of role-play or facilitator ‘scripts’ that are developed and used in artificial or simulated training environments (cf. [46]). Resources based on real-life facilitation and informed by Conversation Analysis could provide facilitators, at different stages and in different roles [4], the opportunity to rehearse different communication practices, obtain feedback on these practices, and critically reflect on their own and others’ communication. Whilst this has yet to occur within Implementation Science, conversation analytic research has been used extensively as a training resource in other fields. For example, the Conversation Analytic Role-play Method (CARM [46];) is a data-driven, immersive, and reflexive approach to communication skills training that has demonstrated success in various contexts (e.g., [47, 48]). CARM moves beyond simulated role-play and allows trainees to view and discuss authentic audio and video data and consider how specific conversational turns and responses lead to desired or undesired outcomes.

In addition to improving the operationalisation of facilitation, utilising Conversation Analysis to understand facilitation in interaction might enable us to better evaluate implementation success. Analysis of interactions (e.g. facilitation meetings) might prove useful not only to operationalise how one might *do* facilitation, but to *evaluate the success* of facilitation and assess the fidelity of interaction-based interventions. For example, Conversation Analysis might enable us to identify the achievement of agreed implementation goals; the uptake and embedding of an innovation into practice; and individual, team and stakeholder engagement, motivation, and ownership of the innovation. Specifically, one of the Implementation Science papers on facilitation discussed in this paper suggested that facilitators want to see recipients ‘bringing up ideas’ (Young et al, 2018). This could be utilised as an indicator of positive engagement, which should be supported and monitored by facilitators as, bringing up ideas, can service as a ‘real-time’ indicator of recipients’ level of engagement. A conversation analytic examination of such an action (i.e. bringing up an idea) would also afford greater insight into (1) how the facilitator and others respond to new ideas and (2) how ideas then translate (or fail to translate) to broader plans for action.

What remains unanswered by alternative research approaches (e.g. interview and observation-based methods), and the specific questions Conversation Analysis can help us understand, include:

- How are the goals of facilitation, as defined in existing i-PARIHS literature (e.g. building relationships, engaging recipients, see Table 2), achieved in conversation?
- What conversational behaviours do facilitators and recipients employ when adopting the i-PARIHS framework, and to what effect?
- Are there interactional behaviours that facilitators and/or recipients adopt without explicit knowledge of their doing so (and, as such, are not described in existing literature)?
- Could an interaction-focussed analysis of facilitation meetings afford access to tacit/implicit behaviours that are, to date, absent from the i-PARIHS literature?
- How does facilitation in the context of i-PARIHS implementation differ from facilitation in other contexts (such as those presented in Table 3)?
- How do facilitators and recipients demonstrate, in their interactions, sensitivity to the other components of the i-PARIHS model (i.e., Innovation, Recipients, and Context?)

Overall, we have put forward Conversation Analysis as a potentially powerful methodology for making sense of the nuances of facilitation, an inherently interactional component of the i-PARIHS framework. Recording facilitation meetings, transcribing the interactions and analysing the sequential behaviours within, would afford the opportunity to answer the above questions, and to operationalise facilitation.

## Conclusion

In this paper, we sought to demonstrate how a conversation analytic approach to examining facilitation of an intervention in healthcare settings might assist in overcoming one of the most common criticisms of the i-PARIHS model: that facilitation, although a critical component of the implementation process, is to-date not adequately operationalised. By examining facilitation in a way that pays respect to its interactional achievement, we put facilitators and recipients in a better position to successfully implement research into practice, and thereby improve healthcare outcomes. Moving forward, our aim is to use this approach to develop resources for users of i-PARIHS to support facilitation and subsequent implementation success. We are currently collecting recordings of facilitation meetings to initiate this programme of work.

## Supplementary information

**Additional file 1.** Jeffersonian transcription symbols.

## Data Availability

Not applicable.

## Abbreviations

i-PARIHS: Integrated Promoting Action on Research Implementation in Health Services
PARIHS: Promoting Action on Research Implementation in Health Services

## Footnotes

1 An ‘extract’ is a section of a transcript chosen by the analyst/author to support a particular claim, used in the same way that interview research includes participant quotes. Inclusion of extracts as evidence is critical in conversation analysis, as they permit a certain degree of ‘check-ability’ of the analysis (i.e., the reader can compare the stated findings with the data).
