# Supplementary material for: Introducing an interactional approach to exploring facilitation as an implementation intervention: examining the utility of Conversation Analysis
Source: Implement Sci Commun. 2020 Nov 4;1:98. doi: 10.1186/s43058-020-00071-z (PMC7640407; doi:10.1186/s43058-020-00071-z)
Supplement: Supplementary file 1 — Additional file 1. Jeffersonian transcription symbols. [file 43058_2020_71_MOESM1_ESM.docx]

| Symbol | Meaning |
| --- | --- |
| () | Transcriber could not hear what was said. |
| (.) | A tiny, noticeable pause. |
| (0.5) | Decimal numbers indicate elapsed time in silence by tenth of seconds. |
| (1) | Whole numbers indicate elapsed time of one whole second. |
| .hhh | The sound of inhalation |
| hhh | The sound of exhalation |
| Word | Underlining indicates emphasis on that word or syllable. |
| WORD | Uppercase indicates especially loud sounds relative to the surrounding talk. |
| 0 0 | Utterances are relatively quieter than surrounding speech. |
| ? | A question mark indicates a rising intonation, less pronounced than upward arrow. |
| ↑ | Shifts into higher pitch. |
| ↓ | Shifts into lower pitch. |
| Wo- | Hyphens mark the abrupt cut-off of the proceeding sounds. |
| >< | Speeded up talk. |
| <> | Slowing down talk. |
| ((smiley voice)) | Words that sound like they have been delivered through a mouth forming a smile. |
